# Mechanical and Biological Interactions of Implants with the Brain and Their Impact on Implant Design

**DOI:** 10.3389/fnins.2016.00011

**Published:** 2016-02-09

**Authors:** Dimiter Prodanov, Jean Delbeke

**Affiliations:** ^1^Department of Environment, Health and Safety, ImecLeuven, Belgium; ^2^Neuroscience Research FlandersLeuven, Belgium; ^3^LCEN3, Department of Neurology, Institute of Neuroscience, Ghent UniversityGhent, Belgium

**Keywords:** neural prostheses, implantable devices, Utah array, Michigan probe, diffusion, blood-brain barrier, micromotions

## Abstract

Neural prostheses have already a long history and yet the cochlear implant remains the only success story about a longterm sensory function restoration. On the other hand, neural implants for deep brain stimulation are gaining acceptance for variety of disorders including Parkinsons disease and obsessive-compulsive disorder. It is anticipated that the progress in the field has been hampered by a combination of technological and biological factors, such as the limited understanding of the longterm behavior of implants, unreliability of devices, biocompatibility of the implants among others. While the field's understanding of the cell biology of interactions at the biotic-abiotic interface has improved, relatively little attention has been paid on the mechanical factors (stress, strain), and hence on the geometry that can modulate it. This focused review summarizes the recent progress in the understanding of the mechanisms of mechanical interaction between the implants and the brain. The review gives an overview of the factors by which the implants interact acutely and chronically with the tissue: blood-brain barrier (BBB) breach, vascular damage, micromotions, diffusion etc. We propose some design constraints to be considered in future studies. Aspects of the chronic cell-implant interaction will be discussed in view of the chronic local inflammation and the ways of modulating it.

## 1. Introduction

The therapeutic use of electrical stimulation of the nervous system is a rapidly growing field already distributed over a wide range of applications, such as Deep Brain Stimulation for Parkinson's disease (Beitz, 2014), tremor, dystonia and obsessive-compulsive disorder (Greenberg et al., 2010); vagus nerve stimulation for epilepsy (Morris et al., 2013), respiratory pacing prostheses; the spinal cord stimulation to control chronic pain (Wolter, 2014) and the auditory neural prostheses, to name just the most broadly accepted treatments. Despite this acceptance there is still much space for progress, for example, by providing bi-directional (i.e., including sensing) interfaces, which would open the path for closed-loop approaches and titration of stimulation. Also, a better stability of invasive brain computer interfaces would directly impact on applications, such as treatment for locked-in patients, complete spinal cord injury and *brachial plexus* injuries, to name but a few.

Electrodes are used in both the central and peripheral nervous systems. The electrodes' shapes are thus often completely different. On the actuator side, there is an abundant literature on *functional electric stimulation* in terms of selectivity, but this problem in not in the scope of the current review. Clearly, some principles and findings in the brain apply to the peripheral nerve and vice versa. However, there are significant differences as well, for example the presence of regeneration of damaged peripheral nerves and its absence in the brain and spinal cord (recent review in Gaudet et al., 2011; Rotshenker, 2011). For example, the brain has a much richer variety of glial cell types. Also, the anatomical and geometrical requirements for electrical stimulation and recording are obviously different in the axially symmetric bundle of axons compared to the more complex distribution of neuronal cells, fibers and synaptic structures in the brain.

On the sensing side, long-term recordings from the human cortex have only been obtained relatively recently (Hochberg et al., 2006; Simeral et al., 2011). A substantial milestone toward the realization of invasive brain computer interface was the clinical availability of the Utah-type of multielectrode array. The first proof of concept was obtained in a pre-clinical study whereby the Bionic (Cyberkinetics, Inc., Foxboro, MA) silicon probe array of 100 microelectrodes implanted in the primary motor cortex of three macaque monkeys (Suner et al., 2005) recorded signals for at least 3 months and up to 1.5 years. This device allowed the animals to control an eight-direction, push-button task. Later, the results of implantation in the primary motor cortex of a tetraplegic patient demonstrated that intended hand motion still modulated cortical spiking patterns 3 years after spinal cord injury. Decoders were created, providing a “neural cursor” with which the patient opened simulated e-mails and operated a TV set. Furthermore, the patient used neural control to open and close a prosthetic hand, and perform rudimentary actions with a multi-jointed robotic arm. These pioneering results suggest that neural prostheses based on intracortical neuronal ensemble spiking activity could provide a valuable new neurotechnology to restore independence for paralyzed humans (Hochberg et al., 2006).

The follow up study of Simeral et al. (2011) focused on critical information obtained from a small group of chronically implanted patients. The main questions were on how long implanted microelectrodes would record useful neural signals, what degree of acquisition and decoding reliably could be reached, and how effective assistive technology based on functional electrical stimulation of paralyzed muscles can be. Five consecutive days of trials performed by a tetraplegic patient 1000 days after implantation of an intracortical microelectrode array demonstrated that such a neural interface system can provide repeatable and accurate control of a computer interface.

Despite these promising case studies, chronically stable interfaces allowing highly selective communication with the human nervous system remain elusive. One of the reasons for this is a limited understanding of the interactions between the implant and host nervous system. Historically, these interactions were lumped in the very broad and therefore vague concept of *biocompatibility*. Unfortunately, the vast majority of publications have looked at biocompatibility only from a binary outcome perspective (i.e., acceptable/rejection) with little or no attention to the detailed causal mechanisms involved. However, better knowledge of these mechanisms is a necessary condition for further progress. Functional neural tissue survival, minimal distance from electrode contact to target and long term stability are essential outcome parameters to be considered in a systematic way. Factors pertaining to various scientific domains (chemistry, cellular biology, physiology, bio-electricity, electrochemistry, anatomy, surgery, microbiology, mechanics) can all affect the final result. Moreover, these factors interact in intricate ways, calling for more attention to second order mechanisms. Shortcomings of the current definition of the bicompatibility have been pointed out by Ratner (2011), who advocates for profiling the tissue response also as function of the scaffold micromechanical properties.

The neuroprosthetic field has come up with many proposals for improving the channel count (for example Lopez et al., 2014) in sensing and decoding as well as in selective stimulation applications without, however, producing implantable devices meeting the necessary specifications in a longterm implantation. Bio-mechanical aspects of implants and their interaction with the nervous system have been sidelined in comparison with aspects of material toxicity, effects of the surface micro-geometry, leaching chemicals, implant sterilization and tolerance to electrical stimulation currents. Interested readers can consult the recent reviews of Gunasekera et al. (2015); Jorfi et al. (2015); Kozai et al. (2015) on these subjects. The present review will, on the other hand, focus on the mechanical interaction between the brain and the implant. We also discuss the mechanisms of neuroinflammation in this perspective.

## 2. Chronic recording *in vivo* studies: current understanding

Metal wire electrodes have been used for acute recordings since the first half of the XX^th^ century. Several types of multichannel electrodes have been developed, including stereotrodes (McNaughton et al., 1983), multiwire arrays, polymer substrate probes (Rousche et al., 2001; Lind et al., 2013), ceramics-based probes (Moxon et al., 2004) and various types of silicon-substrate probes (Wise et al., 1970; Campbell et al., 1991; Jones et al., 1992; Aarts et al., 2008; Musa et al., 2009; Andrei et al., 2012a; Lopez et al., 2014). Electrodes for chronic use have different mechanical properties, resulting in different degrees of invasiveness to the brain tissue.

With the exception of the pioneering work of Goldstein and Salcman (1973) the importance of mechanical factors and their impact on the electrode design has not been recognized in the majority of literature. Recently however, some investigators started exploring different mechanical configurations of implants. There is still much room for improvement of the fabrication technologies because most implants were designed for experimental animal use and were hand made in small numbers.

For example, in a first comparative study, Szarowski et al. (2003) evaluated the tissue response to silicon implants of different sizes and surface characteristics. These authors reported that the final glial scar formation was unaffected by that explored variables, although the initial wound response was different for different array geometries. It should be noted, however, that Szarowski et al. (2003) used two insertion procedures: fast automatic (2 mm/s) and manual (roughly matching by automatic speed). Such fast insertion is expected to cause 4.7 mm dimpling and from contemporary perspective (see Section 4.3, Prodanov et al., 2009; Andrei et al., 2011) and the practice of tetrode recordings, this insertion condition was sub-optimal.

More recently, Ward et al. (2009) compared different implantable commercial microelectrode array configurations. Specifically, NeuroNexus (Michigan) probes, Cyberkinetics (Utah) Silicon and Iridium Oxide arrays, ceramic-based thin-film microelectrode arrays (Drexel), and Tucker-Davis Technologies microwire arrays were evaluated over a 31-day period after implantation in rats. Results demonstrated significant variability within and between microelectrode types especially in terms of the induced gliosis. It is difficult to attribute more than phenomenological weight to this study since the authors investigated complete functional implants with fixed designs.

**Figure 1:**
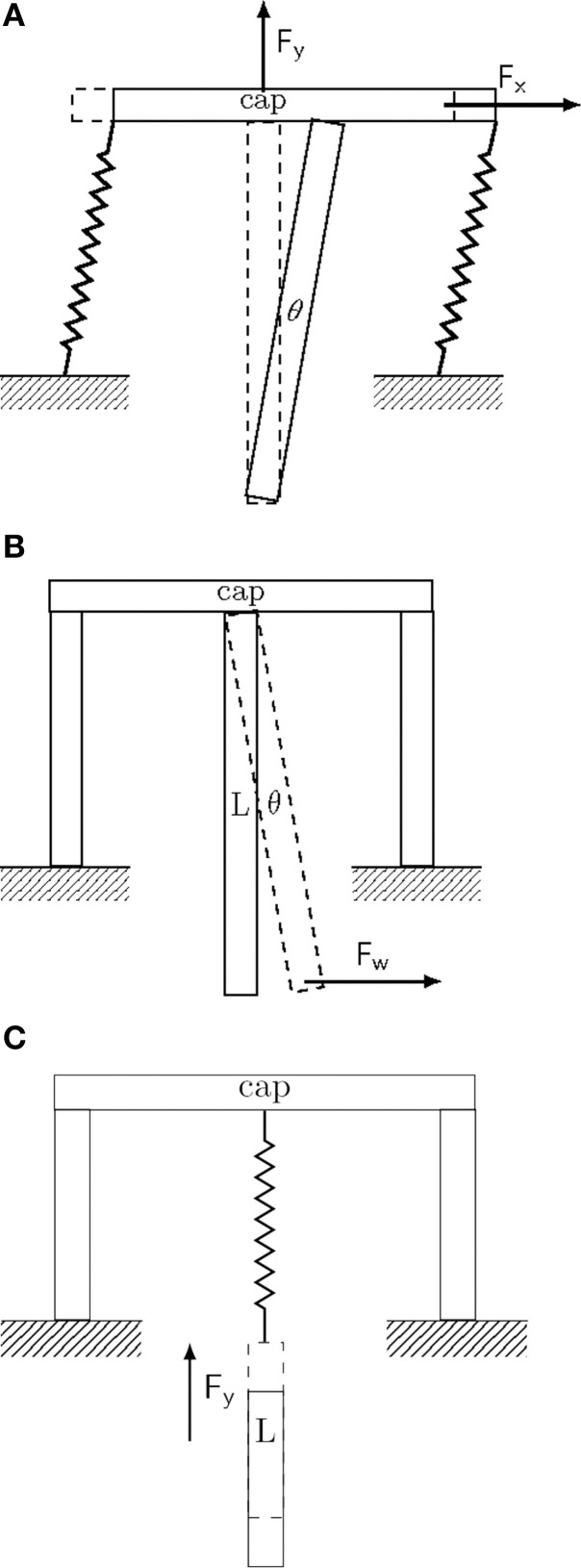
**Conceptual drawing of different implant fixation configurations**. Drawings represent extreme and idealized cases of implant fixation to the skull (marked as *ground*). **(A)** Tethered fixation with semi-flexible attachment. The extent of *XY*-displacement is controlled by the semi-rigid attachment joints. Brain micromotion and body movements result in displacements predominantly in the upper part of the implant along all three spatial directions. Maximal displacement and pressure is attained about the entry point of the implant in the brain (see for example Welkenhuysen et al., 2011). **(B)** Rigid fixation mode. Brain micromotion caused by cardiovascular activity results in displacements predominantly in the lower part of the implant along all three spatial directions. **(C)** Non-tethered fixation mode. Motions of the body result in free displacement about the *X* and *Y*-axes, while the displacement in the *Z*-axis is controlled by the spring constant of the attachment joint.

The examples given above call for critical reappraisal of the experimental design and approaches to assess *biocompatibility* of any given implant. In addition, different permanent fixation modes of the implants as well as the frequent lack of data about the fixation make comparisons between studies difficult (see Figure 1). There is an urgent need for explicit systematic investigations of the mechanisms of failure and their impact on the design parameter space. Important questions are still usually left unanswered in the literature. We could list some here, for example:

- Will a temporary blocking of inflammation after an implantation result in a stable final state ultimately different from the situation when no blocking was applied?
- Is tissue reaction limited in time despite the further presence of the implant or does its progression remain unstable as long as the implant is present?
- Does the observation of an active inflammatory reaction imply that some triggering factor (mechanical or other) is still present?

## 3. Timetable of the implantation effects

Over 100 studies have described stereotypic features of the brain response to microelectrodes that occur irrespective of the type of implant, method of sterilization, species studied, or implantation method (Jorfi et al., 2015). Conventionally, only acute, sub-chronic and chronic periods are discriminated in the literature without agreement on the exact timing. On the other hand, recent studies involving novel methodologies (Potter et al., 2012; Saxena et al., 2013; Xie et al., 2014) demonstrated more granularity in the timing of the described effects.

Recently, Saxena et al. (2013) monitored the status of the blood-brain barrier (BBB) and the consequences of BBB breach on electrode function using non-invasive imaging, electrophysiology, genomic, and histological analyses. Potter et al. (2012) assessed in detail the neuroinflammatory events and BBB integrity following implantation of non-functional planar single-shank array vs. an identical cortical stab injury. Results also provided strong evidence that the acute phase of implant-associated neurodegeneration is correlated with a permeable BBB, while chronic neuronal loss is likely to be the result of endogenous tissue events. In their pioneering study, Xie et al. (2014) used *in vivo* Optical Coherence Tomography (OCT) in order to observe the chronic brain reaction for up to 12 weeks after implantation. This technique features a spatial resolution of 10 μ*m*, up to 1.2 mm imaging depth in the brain tissue. During the first 3 weeks after implantation, the OCT signal of the surrounding tissue increased monotonically. The intensity reached a plateau after 6 weeks post implantation. The increased backscattering intensity from the surrounding tissue was attributed to the increased accumulation of astrocytes around the glass fiber itself. Indirectly, similar effect could be measured about 100 μ*m* from the inserted implant by impedance spectroscopy (McConnell et al., 2009a). Based on these data, the following grouping of the effects of implantation can be proposed:

**Surgical** —Main factors there are the vascular damage and the possible occurrence of hemorrhage (Grand et al., 2010) or electrode breakage and other surgical accidents.
**Acute** —developing in the first 24 h in rodents. Among the factors of primary importance there are the tissue dimpling during insertion, direct local tissue damage, pressure effect due to the implant volume, oedema and the acute vascular damage possibly with hemorrhages and or ischemia.
**Progressive** —between 24 h and 3 weeks in rodents. In this phase, there appears to be a spectrum of effects, related to the sterility of the implantation and environment; the acute brain inflammation with BBB breach, haematom resorption and perhaps infection linked to the implantation and environment sterility. The continuous trauma related to micromotion can also play a role. A cell gap forms around the electrode.
**Sub-chronic** —between 4 and 6 weeks in rodents. In this phase, there appears to be a mix of effects, related to tissue remodeling. Chronic inflammation and chronic vascular damage dominate. A neuronal cell gap could develop around the electrode (Section 3.2).
**Chronic** —developing between 6 to 12 weeks in rodents. In the chronic phase, neuronal migration becomes an important additional restauration mechanism whereby functional neurons could reenter the neural cell gap. Electrode failure has often been attributed to the traumatic injury resulting from insertion and a long-term foreign body response to the implant (Turner et al., 1999; Holecko et al., 2005; Rennaker et al., 2005; Ward et al., 2009). As discussed in Section 7.1, mechanical factors participate extensively in the chronic activation of the tissue reaction and thus to the distance maintained chronically between the electrode and the neurones of interest.
**Steady (persistent)** —a chronic stable modified state developing after 12 weeks in rodents. The most important properties, in our opinion, of the persistent response are comprehensively discussed in Section 7 of the review.

It should be noted that this timing is derived from rats studies and, therefore, can serve only as a preliminary guideline to the situation in men or primates due to differences in the immune response and the rate of metabolism.

### 3.1. Consitituents of the neuroinflammation process

Chronic foreign body response emerges as a complex phenomenon resulting from multiple, interconnected yet parallel processes.

Even when the neural tissue itself remains untouched, as in a cuff wrapped around a peripheral nerve, tissue destruction during implantation and the presence of foreign material (Szarowski et al., 2003) induce the release of cytokines responsible for triggering an inflammatory process (Vince et al., 2005a,b) and neo-vascularization (Thil et al., 2005). This reaction includes an initial edema typically related to breaching of the blood-neural barrier. Cytokines also contribute in maintaining the BBB breach (Abbott et al., 2006; Saxena et al., 2013), which in turn seems to feed a persistent inflammation and enhanced BBB permeability that was found more variable at 3 months than at 2 and 4 weeks (Winslow and Tresco, 2010).

Some secreted *cytokines* can promote while others can inhibit glial scar formation. Interested readers are directed to the review of Stichel and Müller (1998). We note here only the role of interleukin 1 (IL-1) and interleukin 6 (IL-6) as possibly main promoters of astrogliosis.

Attachment and clustering of *microglia* on the implant surface is a well documented phenomenon in all *in vivo* studies. Clustering and attachment of microglia to the implant has been demonstrated for example by Winn et al. (1989); Menei et al. (1994). This attachment is thought to be mediated by the adsorption of albumin on the implant surface or due to the release of chemo-attractants by serum factors, such as monocytes chemotactic protein-1 (MCP1) and macrophage inflammatory protein (MIP-1) at injury sites (Saadoun et al., 2005). From spiny shape the activated microgila shifts to ameboid shape bearing multiple phagocytic vesicles. It upregulates its lytic enzymes and starts expressing MHC I and II surface molecules. Upon activation microglia and macrophages share most phenotypical markers and can exert similar effector functions. Microglia can secrete cytokines, such as IL-1, IL-6, and MCP-1, which are able to further recruit other microglial cells and macrophages. The number of ED-1 (a specific cellular marker for activation of rat macrophages) labeled cells increases progressively for several days after implantation, suggesting the potential recruitment of peripheral blood-borne macrophages, as well as a transformation of endogenous microglia into brain macrophages (Barrese et al., 2013). The numbers of microglial cells initially increase sharply in the implanted region during the recovery phase reflecting a local microglial proliferation but the increase does not continue further in the chronic phase.

**Figure 2:**
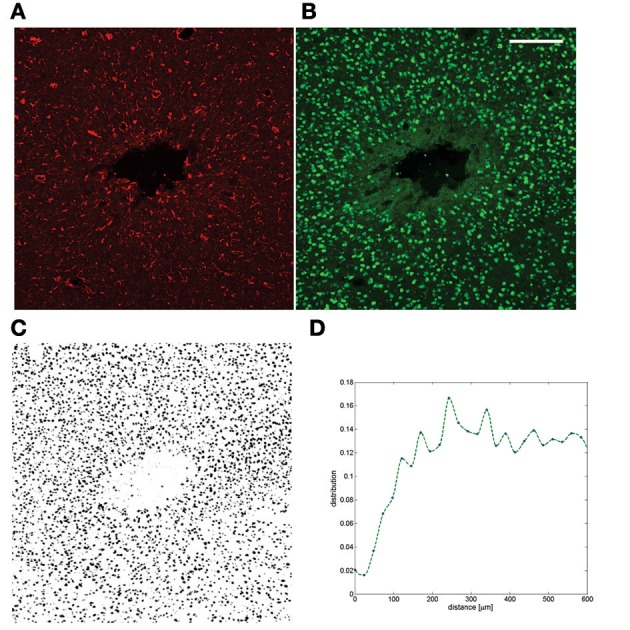
**Example of a chronic neuroinflammatory response to implantation of a silicon probe**. **(A)** GFAP staining after 6 weeks of implantation in tethered configuration; **(B)** NeuN from the same section **(C)** The full NeuN image was thresholded and the area fraction is plotted as a function of distance (Prodanov and Verstreken, 2012) to the insertion track **(D)**. The neuronal cell gap is clearly visible. The dataset was published in Welkenhuysen (2011). Scale bar—200 μ*m*.

The reactive *astrocytes* form a dense web of interdigitated processes around the implant (Turner et al., 1999), which fills the space occupied by the dead or dying cells. The activated astrocytes overexpress the Glial Fibrillary Acidic Protein (GFAP). The resulting state of the tissues surrounding implanted electrodes is typically characterized by the presence of a fibrous encapsulation of the foreign material and the absence or reduced density of functional neural cells in the immediate proximity of the implanted electrodes (Edell et al., 1992) referred to as the neural cell gap (see Figure 2). In the brain, the astrocytes appear to be a major actor but by far not the only cells involved in the still poorly understood neuroglial defense mechanisms (Polikov et al., 2005).

Astrogliosis and scar formation promote wound closure, neuronal protection, BBB repair and restriction of CNS inflammation (review Sofroniew, 2015). Nevertheless, under specific circumstances, astrogliosis has the potential to lead to harmful effects, such as exacerbating inflammation.

Morphology and functional properties of the *extracellular matrix* in the brain are modified by laminin, fibronectin, tenascin C and proteoglycans produced by activated cells. Normal diffusion paths in reactive astrogliosis are significantly affected (Roitbak and Syková, 1999). The glial scar involves gross cellular and molecular rearrangement of the tissue components, which forms inhibitive environment for the regeneration of nerve fibers. Components of the extracellular matrix, such as proteoglycans, appear to be main factors for this inhibition (review in Fitch and Silver, 2008). The extracellular matrix components may be secreted by reactive astrocytes, oligodendrocyte precursors, microglia/macrophages and eventually by meningeal cells. The lesion and resulting reactive processes induce a matrix accumulation that strongly resembles the juvenile-type of meshwork previously observed during early nervous system development (Zimmermann and Dours-Zimmermann, 2008). Neural cells and the extracellular matrix can either quickly reach a new stable state or the reaction mechanisms can remain active in a chronic struggle to adapt (Gaudet et al., 2011). In the latter case, the presence of a persistent aggression can be suspected, perhaps involving a persistent BBB breach (Saxena et al., 2013).

### 3.2. The neuronal gap

Concurrent with the glial scar formation, neuronal density within the recording radius of the microelectrodes decreases (see for example Grand et al., 2010). Some authors describe a kill zone9D in the vicinity of the implanted probe (Biran et al., 2007) (see Figure 2). This leads to a decrease of single-units distinguishable in recordings (Edell et al., 1992; Turner et al., 1999; Kim et al., 2004; Biran et al., 2005; Purcell et al., 2009). It should be noted that this picture is not observed in all electrode sites. Notably, some sites exhibit very stable performance even if the number of distinguishable single units varies from session to session (Liu et al., 1999).

The loss of neuronal cells observed *in vivo* is not necessarily a consequence of elevated glial activation. Some authors (Grand et al., 2010) explicitly link loss of signal to hemorrhages around recording sites. Also it is suggestive to note that transmission electron microscopy shows normal synapses within a few micrometers from implanted polymer capsules (Winn et al., 1989), which at first glance seems inconsistent with the decrease in neurofilament densities described around implanted rigid electrodes (Biran et al., 2005). On the other hand, such observation may be explained by the lack of relative displacement of such small capsules during motion of the animal (see Section 7.1). As already observed in literature, tethering of the implant (Kim et al., 2004; Biran et al., 2007; Thelin et al., 2011) and micromotions are indeed anatomy-linked parameters that further activate the inflammatory process.

The origin of the neural cell gap is still unclear with the possibility that functional neural cells are simply pushed away by the encapsulation developing between them and the electrodes (Georges et al., 2006). Some authors have suggested that many neurons around the electrodes die shortly after implantation (Edell et al., 1992; Biran et al., 2005). However, a more progressive loss of neurons, dendrites and synapses *progressive degeneration of nerve fibers and synapses* could result from a persistent local chronic inflammation (McConnell et al., 2009b).

It is known that some of the released neurokins induce neurodegeneration (Carson et al., 2006). The gap between electrode and functional neurons can thus result from a loss of neurons that have undergone apoptosis (Lull and Block, 2010; Gaudet et al., 2011) or from locally non-functional synapses (Winslow and Tresco, 2010). Demyelination and the resulting conduction block are yet another possibilities.

On a different note caspases have been also implicated in this process. Caspase-1 (i.e., Interleukin 1β converting enzyme) is known to play a key role in both inflammation and programmed cell death, particularly in stroke and neurodegenerative diseases. Recently, Kozai et al. (2014) demonstrated improved electrophysiological recording in caspase-1 knock out mice. Obtained recordings showed significantly improved single-unit recording performance (yield and signal to noise ratio) of the knock out mice compared to wild type mice over the course of up to 6 months for the majority of the depth. The higher yield is supported by the improved neuronal survival in the knock out mice.

### 3.3. Blood-brain barrier properties

The term BBB, describes the phenomenon that was first observed by Paul Ehrlich, who noted that during intravital staining the brain showed little or no coloration. Later the Berlin physician Lewandowski injected sodium ferrocyanide into the CNS, from which he concluded that the “capillary wall must block the entrance of certain molecules” not normally present in the blood (review in Owens et al., 2008).

Subsequent research has established that in unison with pericytes, astrocytes, and microglia, BBB separates components of the circulating blood from neurons. Physiologically, BBB refers to the vascular segment of the capillaries that regulate diffusion of solutes, whereas in an inflammatory response, the term refers to the postcapillary venules, that is, the vessels from which leukocytes migrate into the CNS, which are distinct vascular segments (Owens et al., 2008). Moreover, the BBB maintains the chemical composition of the neuronal milieu, which is required for the proper functioning of neuronal circuits, synaptic transmission, synaptic remodeling, angiogenesis, and neurogenesis in the adult brain. Endothelium, the site of anatomical BBB, neurons, and non-neuronal cells (e.g., pericytes, astrocytes, and microglia) together form a functional unit, currently denoted as *neurovascular unit*. Vascular cells, i.e., endothelium and pericytes, can directly affect neuronal and synaptic functions through changes in the blood flow, the BBB permeability, altered secretion of trophic factors and matrix molecules, change in the expression of vascular receptors, or induction of ectoenzymes (review in Zlokovic, 2008).

As highlighted by Owens et al. (2008) the barrier concept is more applicable to solute entry, the neuroinflammatory relevance of which relates more to edema than to cellular migration. The process of leukocyte entry into the CNS parenchyma is controlled by different cellular components at the level of postcapillary venules. In order to reach the CNS parenchyma, leukocytes need to perform **two** differently regulated steps: first, to cross the vascular wall, and second, to traverse the glia limitans. Current research indicates that these steps are controlled by different mechanisms. It seems that activated lymphocytes regularly penetrate the endothelial barrier for immunosurveillance of the CNS, but only upon penetration of the glia limitans and infiltration of the CNS parenchyma do leukocytes come into direct contact with the parenchyma, which leads to clinical symptoms.

## 4. Design constraints

### 4.1. Accessibility of the implantation site

Accessibility, as determined by the anatomical region that will receive the implant and the geometry and position requirements of the device, directly determines the invasiveness of the implantation procedure and even its feasibility. This can be influenced by the health state of individual patients as is the rule for cochlear implants (Nadol, 1984). Subcutaneous devices present little surgical challenge. However, in addition to local scar and infection risks there is always the possibility of skin necrosis through an increased local pressure (Ishida et al., 1997). Trephination (i.e., breaching the skull) represents a significant increment in invasiveness (Anderson et al., 2008). One step further, penetrating the *dura mater* leads to the possibility of cerebro-spinal fluid leakage (Waziri et al., 2009) which will require revision surgery. Finally, devices that are difficult to place or to fixate will require longer surgical procedures involving larger risks.

Among other geometrical concerns, formation of poorly perfused pockets that could favor infections must be avoided. By visual guidance, the implantation surgery can preserve larger vessels but this is not possible with capillary vessels when the implant is a multiple needle electrode array. For example, in the mouse brain neurones are never further than 15 μ*m* from a microvessel (Tsai et al., 2009). Clearly, even small electrodes will damage blood vessels at insertion, sometimes with catastrophic local consequences (Grand et al., 2010).

### 4.2. Viscoelastic properties of the brain

Material stiffness has been recognized as an important design feature for implants (Georges et al., 2006). However, mechanical characteristics of organic tissues in general and the brain in particular are not purely elastic. The strain (ϵ) rate significantly affects the resulting stress force (σ) (Bjornsson et al., 2006; Welkenhuysen, 2011; Andrei et al., 2012a). In order to account for this, a viscous component (η) must thus be added to the elasticity modulus (*s*) resulting in a complex strain-stress tensor. Strain and stress are closely related to the alternative parameters “shear” (measured in radians) and shear stress force respectively.

Modeling approaches have been proposed to characterize these parameters (Rashid et al., 2012) but these still do not include issues, such as the long-term changes in mechanical properties of brain tissue. Indeed, after implantation, the device-tissue interface shear modulus estimated according to a 2nd order viscoelastic model increases from 0.5–2.6 kPa to 25.7–59.3 kPa after 4 weeks and then decreases to 0.8–7.9 kPa after 6 to 8 weeks (Sridharan et al., 2013). The authors also report the corresponding elastic modulus value of 4.1–7.8 kPa on the day of implantation, 24–44.9 kPa after 4 weeks and 6.8–33.3 kPa at 6–8 weeks. These estimates suggest that the brain tissue surrounding the microelectrode evolves from a stiff matrix with maximal shear and elastic moduli after 4 weeks of implantation to a composite of two different layers with different mechanical properties—a stiff compact inner layer surrounded by softer brain tissue. It is anticipated that there are two scenarios where viscoelasticity plays a major role: during device insertion and during the indwelling period in combination with micromotions (see Section 7.1).

### 4.3. Insertion force and tissue dimpling

Insertion of an electrode into brain results in a tissue deformation called dimpling by the action of mechanical forces. When the electrode shaft is not stiff enough to resist the applied force it can buckle and then implantation is impossible. Below that critical limit the magnitude of insertion force and resulting dimpling are closely related and they can damage blood vessels and neuronal tissue as illustrated by Bjornsson et al. (2006). These factors can lead to irreversible localized traumatic brain injury (TBI) and poor detection of neuronal activity during electrophysiological measurements (Rennaker et al., 2005).

The extent of brain tissue damage is related to the implant geometry, for example tip angle, cross sectional area and overall configuration (wire electrode, needle, array), mechanical constants, and the applied insertion speed (see Section 4.2) (Jensen et al., 2006; Hosseini et al., 2007; Sharp et al., 2009; Casanova et al., 2014). The insertion force was only recently measured in some experiments mapping the range of possible values for different electrode geometries. Hosseini et al. (2007) measured the insertion forces for slender probes made from silicon, glass, tungsten and polyimide. The *in vivo* insertion forces through the dura was measured to be 41 ± 25.5 mN for rats and about 140 mN for monkeys, with tip angle 17°, width 120 μ*m* and thickness 100 μ*m* (Hosseini et al., 2007). An *in vivo* rat pia membrane penetration stress between to 0.04 and 0.12 *mN*∕μ*m*^2^ was found for Michigan microelectrodes with cross-sectional area of 900 and 1200 μ*m*^2^ and opening angle 60° (Najafi et al., 1990).

Recently, Casanova et al. (2014) found a surface dimpling of 650, 740, and 900 μ*m* for insertion speeds of 0.2, 2, and 10 mm/s during insertion of sharps needles in the brain, respectively. In another example, using a 200 μ*m* wide probe with a tip angle of 90° inserted at 10 μ*m*∕*s*, Welkenhuysen et al. (2011) measured an insertion force of 98 mN when the dura was punctured and only 4.14 mN when the dura was removed before insertion. These values reduce to respectively 75 and 2.57 mN if the probe width is reduced to 100 μ*m*. For insertion speeds of 100 μ*m*∕*s*, the authors measured 180 and 2.75 mN for the 200 μ*m* probe reducing to 87 mN and 1.51 mN with the 100 μ*m* devices. Harris et al. (2011) reported insertion forces of 2.30 ± 0.38*mN* with 4.7 mm dimpling and at 8 weeks using devices of 100 μ*m* in thickness, 200 width with and 45° tip angle, inserted at 2 mm/s, The study demonstrated an increased cell density at the microelectrode tissue interface without tissue necrosis or excessive gliosis. Andrei et al. (2012a) demonstrated that a bevel shaped sharp silicon tip facilitates insertion of a 1 cm probe with shank made of polyimide, yielding a penetration force of 1 mN through the dura.

**Figure 3:**
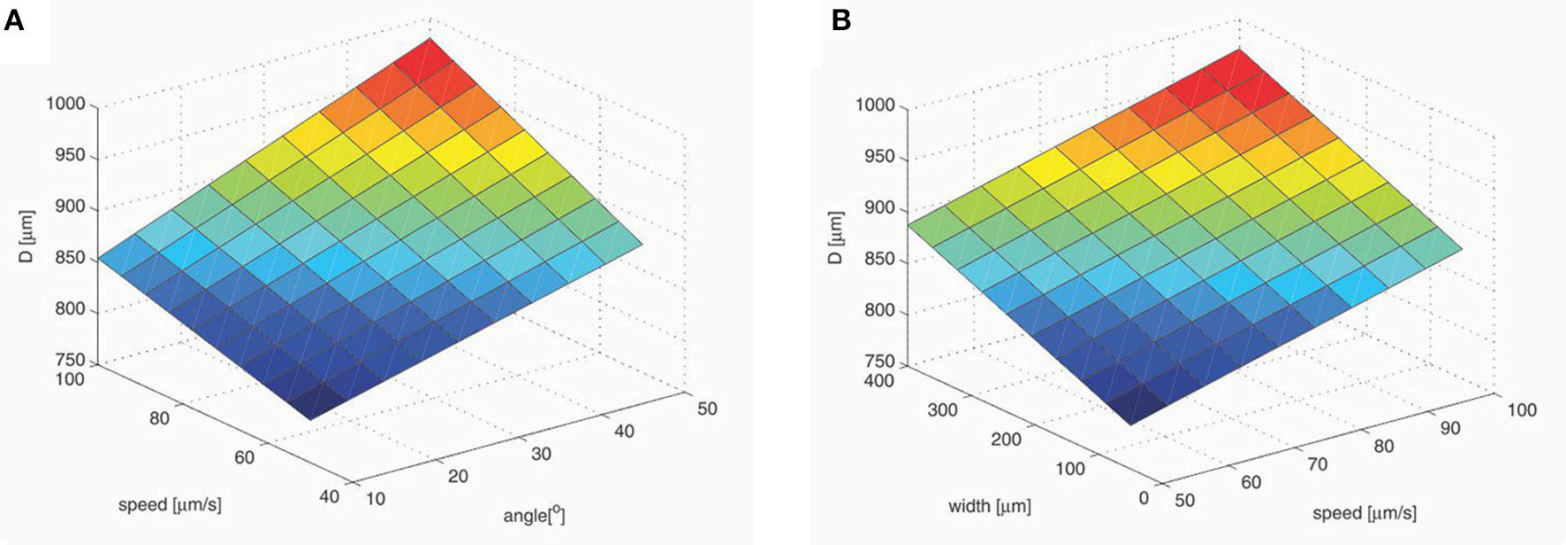
**Tissue dimpling during insertion**. **(A)** Shaft width is fixed at 200 μ*m*; **(B)** Tip angle is fixed at 30°. Values are calculated according to Andrei et al. (2012b). The brain tissue dimpling is given by the following formula : *D*[μ*m*] = 664.47 + 0.2715 · *w* + 0.0216 · *a* · *s* + 1.0339 · *s* + 1.0096 · *a*, w—shaft width in μ*m*, a—tip angle in °, s—insertion speed in μ*ms*^−1^. The color map corresponds to intensity in vertical direction (e.g., blue—low, red—high).

Different tip and shank geometries as well as various insertion speeds have been tested in other studies in order to evaluate insertion forces and tissue dimpling (Jensen et al., 2006; Hosseini et al., 2007; Sharp et al., 2009). Most of these studies evaluated the contributions of only one or two parameters at a time, while fixing the rest. Interactions between different parameters were investigated by Andrei et al. (2012a). The authors proposed a statistical model predicting the force response in the usual design space. The model is reproduced here in Figure 3. This model estimates tissue dimpling resulting from the implantation of an electrode with given tip angle, width, thickness and insertion speed (Andrei et al., 2012b).

The study of Rennaker et al. (2005) provides initial evidence that mechanical insertion devices, which prevent cortical compression, increase electrode recording longevity. Rennaker et al. (2005) compared two insertion techniques (mechanical and manual) for chronically implanting wire multielectrode arrays in layer IV of primary auditory cortex. The implants consisted of a 2 × 7 arrays of 50 μ*m* diameter tungsten wire spaced 250 μ*m* center-to-center. The implants used a connector separated from the skull cap, thus limiting pulling forces during donning and doffing. Researchers constructed a special mechanical insertion device, capable of rapidly inserting the electrode array without visible compression of the brain. Both techniques resulted in a similar number of active channels directly following surgery with a mean signal-to-noise ratio of approximately 4.5. Over 60% of the animals implanted with the mechanical insertion device had driven activity at week 6 whereas none of the animals with manually inserted arrays exhibited functional responses after 3 weeks. In these experiments, insertion speeds were much higher than the forces used for slender arrays (e.g., about 1.5 m/s), therefore the brain tissue behaved like a non-deformable solid. Accordingly, high speed imaging did not show surface deformation. On the other hand, the manual insertion procedure used a micro-manipulator resulting in giving speeds on 500 μ*m*∕*s* resulting in dimpling of 2–3 mm, which is consistent with the study of Andrei et al. (2012b).

The study of Andrei et al. (2012b) suggests that relatively fast insertion speeds must be avoided due to excessive dimpling. This was already recognized a long time ago in the implantation method of “tetrodes,” where the electrode insertion only reaches the final depth in the course of days. In contrast, fast insertion protocols, such as used for Utah arrays are still used. This brings about the important question about the consistency of the measurement conditions reported in literature.

Maybe very slow and very fast insertions are favorable while there is an intermediate insertion speed range that should be avoided. Stress distribution at the electrode tip includes shear, tensile, and compressive stress components. Cutting is often associated with shear failure, however other modes of failure may also occur in tissues. Viscoelastic extracellular matrix fibers may be stretched and fall in tension in a rate-dependent manner (Casanova et al., 2014). Also discrete cells may shift or simply move out of the electrode pathway. In this case, slower insertion velocities may allow more tissue accommodation.

In addition, friction stress (product of pre-stress and friction coefficient) at the needle-tissue interface relates the amount of tissue contact with dimpling (Sharp et al., 2009). The ACREO silicon electrodes (4° opening angle, 18 shafts) have been compared with single-shaft tungsten electrodes (3 and 10°, Jensen et al., 2006). The authors consistently observed drag forces during the retraction phase which could be eliminated by pretreatment with hydrophobic (silane) or hydrophilic (piranha) agents. For the ACREO electrodes with 5 and 8 shafts, the maximal penetration force were respectively (2.42±0.77*mN*) and (2.04 ± 0.77*mN*). The cross-sectional area of the Michigan and the ACREO electrodes are comparable, therefore the difference in opening angles of the two electrodes may explain the differences in penetration forces. Such assumption is further supported by observations by Edell et al. (1992) that penetration of the dura was found to be more difficult with electrodes that had an opening angle larger than 40–50°, whereas the electrodes with opening angles less than 20° could penetrate the dura without causing any dimpling (Edell et al., 1992). In another study, Andrei et al. (2012a) demonstrate beneficial effects of reducing the friction between the implant and the surrounding tissue by coating the silicon electrode shanks with Parylene C. It is also interesting to note that local collagenase treatment also reduces insertion forces during slow (10 um/s) approach by almost 40% (4.04 ± 2.03 mN vs. 2.36 *pm* 1.17 mN) (Paralikar and Clement, 2008).

Specific surgical approaches to brain structures can also reduce insertion forces. Hence, quite unlike the commonly used approaches, the wires inserted from the white matter side, thus avoiding mechanical pressure on the dura and pia mater during penetration, only minimally disturb the cortical recording site. Hence, Krüger et al. (2010) implanted a brush of 64 microwires chronically at a slanted angle in the ventral premotor cortex of a macaque monkey. By this approach isolated potentials and multiunit activity could be recorded for more than 7 years in about one-third of the electrodes. The indirect insertion method also provided an excellent stability within every recording session, and in some cases even allowed recording from the same neurons for several years. Histological examination of the implanted brain region showed only a very marginal damage to the recording area. Notably, only spotty and localized gliosis.

*In vivo* imaging has shown that the tissue strain surrounding the probe remains hours after insertion (Kozai et al., 2012), though it is unclear how much of that is due to release in friction tension between the tissue and the implant or due to edema, inflammation swelling, or impaired blood flow induced change in intracranial pressure.

### 4.4. Density mismatch

Metals have high mass densities for example tungsten—19.25 *g*∕*cm*^3^, stainless steel—8 *g*∕*cm*^3^, and silicon 2.33*g*∕*cm*^3^, which sharply contrast with the density of brain tissue: 1.045 *g*∕*cm*^3^ (DiResta et al., 1991), or of physiologic fluid: 1.0063−1.0075*g*∕*cm*^3^ and the very low value of 0.925−0.970*g*∕*cm*^3^ measured in adipose tissue of cadavers (Martin et al., 1994). These observations lead some authors to posit that density or specific gravity mismatch between tissue and neural probes is a determining factor for glial scarring (Lind et al., 2013).

It can be argued that a more dynamic view on the matter is of import. It should be noted that during body motion such density mismatches will induce torque and tangential displacements not present at rest. Therefore, it is the asymmetry of the inertial tensor of the implant-tissue system and not only the density mismatch that will result in relative displacements during body and head movements.

Any implant that is anchored to the skull and in chronic contact with meningeal tissue will have a higher level of tissue reactivity than the same material completely implanted within brain tissue (Kim et al., 2004). This points to the fact that tethering, as illustrated in Figure 1, amplifies the inertial effects. Obviously, density is important and bears on other factors as well. For example the size of an implant can modify the average density of that implant. Similarly, large implants will see an average of the densities of various tissue components (blood vessels, scar tissue, soft brain tissue as well as other structures including the *pia mater*), which would not be the case for small devices. The encapsulation tissue formed in reaction to the implant could play a major role as a progressive interface between device and plain brain tissue. Clearly, more experimental work needs to be done about these issues.

### 4.5. Apparent recording site impedance

Electrode impedance is often thought to reflect the presence of a shielding encapsulation reducing stimulation as well as recording electrode efficiency (McConnell et al., 2009a) because of the higher resistivity of this scar tissue (Grill and Mortimer, 1994). However, taking into account the serial resistive component and the circuit-shorting component, a divider network is formed that applied a constant reduction factor as long as all resistivities change in the same proportion and the electrodes are connected to high input impedance amplifiers or current controlled stimulators. In an homogeneous volume conductor and at distance of the source, this translates in a bipolarly recorded potential *V* according the following equation (Woodbury, 1960):

$$V=\frac{abI\rho \cos\phi }{4\pi d^{3}}$$

where *a* and *b* are respectively the source and the recording dipole lengths and *d* is the distance between the dipoles, while ϕ is the solid angle and ρ is the resistivity.

Of course, the situation is much more complex at short distance and in a realistic inhomogeneous volume. However, the formula shows that distance is the culprit rather the tissue impedance. This is also true for current sources. Sohal et al. (2014). Hence, SNR measurements were not found to be related to 1 kHz *in vivo* impedance in both rodents and non-human primates for a variety of microelectrodes (Suner et al., 2005; Ward et al., 2009).

It should be noted that the prevailing tradition of using site impedance as a predictive criterion originates in the use of wire electrodes. There degradation of the wire insulation obviously results in a drop in the electrode impedance. In the case of wire electrodes, low impedance values can also be observed because of poor insulation, cracks in insulation/recording surface, and insulation delamination (Prasad et al., 2014). Such manufacturing problems aggravated by the implantation can lead to limited performances, poor yield and electrode failure that are not correlated with inflammatory events and still yield low electrode electrical impedances. An opposite situation can be indicated for planar electrode arrays, where the interface impedance increases in the case of corrosion of the electrode contact surface. On a different note, tissue impedance measurements can be interpreted correctly only using 4 points measurement configuration which is frequently not the case in various studies.

**Figure 4:**
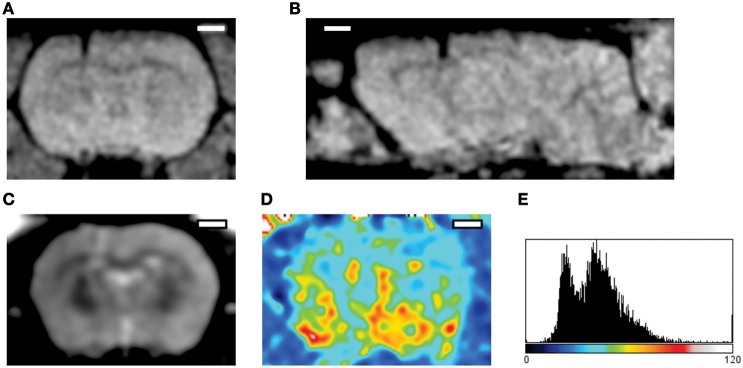
**Imaging of the acute post-implantation phase**. The MRI datasets were acquired 24 h following implantation of a silicon probe in the rat cortex (Prodanov et al., 2009). **(A,B)** FLASH 3D sequence. Parameters: echo time 12 ms inversion time 100 ms 30° flip. **(A)** Coronal projection, **(B)** Sagittal projection across the implantation site; The images exhibit some hypermagnetic signal around the implant artifact (hypomagnetic dark shade). **(C)** Multi Spin Multi Echo (MSME) sequence, for every coronal plane—8 echo times every 10 ms **(D)** T2 relaxation map computed from the sequence; implantation region (left) exhibits higher T2 times that the contralateral cortex. **(E)** Distribution of T2 times, abscissa—ms. T2 latency times follow bimodal distribution indicating the presence of water (higher peak) and solid tissue (lower peak) (Qiao et al., 2001). Scale bars—2 mm.

Similarly, an acute highly conductive edema increasing the distance between electrode and target reduces the recorded amplitudes much more than high resistivity fibrous tissue will later do. Hence, the common observation that both the recorded amplitude and the electrical impedance increase in the initial period after implantation. Such a trend has consistently been observed in animals with 1 kHz impedances progressively increased to a maximum reached at approximately 7 days post-implant (Williams et al., 2007). To further illustrate this dynamics we present brain imaging data following implantation of silicon probes (Prodanov et al., 2009; Figure 4).

Changes in the high frequency region of complex impedance spectroscopy (McConnell et al., 2009a) suggest the presence of edema with progressive infiltration of reactive cells in close proximity to the electrode site within a few days post-implant. If interpreted correctly, impedance measurements can thus be a useful monitoring instrument but there are many possible sources of error including very high interface values at implantation, effect of blood flow, local pressure, movements and the possible confusion between contact interface and tissue resistivity.

## 5. Phenomenology of failure mode studies

The recording longevity of implanted electrodes is highly variable (see for example for wire electrodes—Liu et al., 1999; Nicolelis et al., 2003 and for silicon-based electrodes and multi-wire arrays—Ward et al., 2009). On the other hand, failure mode testing still appears to be very fragmentary in animal studies. This situation limits the translational potential for human neural prosthetic applications. More recently, Jorfi et al. (2015) outlined a number of failure modes likely to affect chronic recording stability and quality:

1. direct mechanical damage of the electrode;
2. corrosion of electrical contacts;
3. degradation of passivation layers and insulating coatings;
4. the neuro-inflammatory response of the brain against chronically implanted devices.

### 5.1. Utah arrays

Utah arrays have been developed as electrodes for cortical visual prostheses (review in Schwartz, 2004). In an attempt to use the array for chronic recording in a human subject (Hochberg et al., 2006) reported failure 6.5 months after implantation due to physical short circuit ground in the electrodes, cable and/or the connector. In the same study in a 55-year-old second human subject an abrupt signal loss due to unexplained technical problems did put an end to 10 months of successful recording.

Using a regular array of 100 microelectrodes, Suner et al. (2005) recorded from the primary motor cortex (MI) of monkeys for at least 3 months and up to 1.5 years. These authors implanted Bionic (Cyberkinetics, Inc., Foxboro, MA) silicon probe arrays in MI of three Macaque monkeys. Neural signals were recorded during performance of an eight-direction, push-button task. Recording reliability was evaluated for 18, 35, or 51 sessions distributed over 83, 179, and 569 days after implantation, respectively, using qualitative and quantitative measures. Neural waveform shape varied between, but not within days in all animals, suggesting a shifting population of recorded neurons over time. Arm-movement related modulation was common and 66% of all recorded neurons were tuned to reach direction. In rhesus macaque monkeys 57, 43, and 39% of original units recorded with Utah arrays were stable for 7, 10, and 15 days respectively as demonstrated on average spike waveforms and interspike interval histograms (Dickey et al., 2009).

Barrese et al. (2013) also investigated longterm failure modes of cortical Utah arrays in non-human primates and demonstrated that most failures (56%) occurred within a year of implantation, with acute mechanical failures being the most common class (48%), largely because of connector issues (83%). Among grossly observable biological failures (24%), a progressive meningeal reaction that separated the array from the parenchyma was most prevalent (14.5%). In the absence of acute interruptions, electrode recordings showed a slow progressive decline in spike amplitude, noise amplitude, and number of viable channels that predicted complete signal loss by about 8 years. Impedance measurements showed systematic early increases, which did not appear to affect recording quality and was followed by a slow decline over years.

In rats, Nolta et al. (2015) studied the performance of Utah Arrays for up to 12 weeks and observed that the foreign body reaction was characterized by a persistent inflammation with expression of typical biomarkers, including presumptive activated macrophages and activated microglia, astrogliosis, and plasma proteins indicative of BBB disruption. Neuronal process distribution was reduced. However, unlike what has been described for recording electrodes that create only a single penetrating injury, a substantial brain tissue loss, generally in the shape of a pyramidal lesion cavity, was observed at the implantation site. Such lesions were also observed in stab wounded animals indicating that the damage was caused by vascular disruption at the time of implantation.

### 5.2. Wire arrays

As early as 1974, Burns et al. (1974) observed a progressive decline in unit recordings in cat cerebral cortex after implantation, with only 8% of the electrodes functioning after 5 months. Results of Williams et al. (1999) with multiwire array singles out two groups of microelectrodes—a group characterized by a rapid deterioration of the SNR and the number of recordable units while another group demonstrated stable performances after an initial rapid but limited decline. The same study reported loosening of skull cap along with medical complications leading to failure within 15–25 weeks of implantation. Liu et al. (1999) reported that the signal from implanted *Ir* wire electrodes are unstable during the acute phases of tissue remodeling, and thereafter experience a continual decrease in recording ability over the ensuing months.

More recently, Freire et al. (2011) evaluated the implantation of tungsten wire microarrays in the motor cortex of rats with weekly recording sessions for 1–6 months. Exhaustive assessment included metabolic markers, inflammatory response, immediate-early gene (IEG) expression, cytoskeletal integrity and apoptotic profiles. The implanted tissue appeared to be histologically, structurally, and metabolically well-preserved; the electrode implantation did not affect the normal physiology of the implanted tissue, as indicated by IEG reactivity; despite a small inflammatory response and gliosis at the implanted sites, cell death was minimal after the multielectrode implantation. However, these authors also observed a significant decay of the number of recorded neurons over the studied 6 month period.

Prasad et al. (2014) performed a comprehensive abiotic-biotic characterization of Pt/Ir arrays in 12 rats implanted for periods ranging from 1 week up to 6 months. Even before implantation, they observed significant structural variations such as irregular insulation, cracks in insulation or recording surface, and insulation delamination. Delamination and cracking of insulation were present in almost all electrodes post-implantation. These changes altered the electrochemical surface area of the electrodes and the electrical leakage pathways resulted in declining impedance values long-term. Overall, this impedance reduction corresponded to a poor electrode functional performance. Obtained results suggested that manufacturing variability and insulation material are important factor contributing to electrode failure. Biotic characterization from the same study showed that poor electrode performance was not correlated with microglial activation except for intraparenchymal bleeding, which was evident macroscopically in some rats and revealed microscopically by intense ferritin immunoreactivity in microglia/macrophages. Thus, intraparenchymal bleeding, suboptimal electrode fabrication, and insulation delamination were identified as the major factors contributing to electrode failure.

### 5.3. Silicon probes

Many authors have worked on the deposition of multichannel miniature and microelectrodes electrodes on various substrates, including polymer substrate probes (Rousche et al., 2001; Lind et al., 2013), ceramics-based probes (Moxon et al., 2004) and various types of silicon-substrate probes (Wise et al., 1970; Campbell et al., 1991; Jones et al., 1992; Aarts et al., 2008; Musa et al., 2009; Andrei et al., 2012a; Lopez et al., 2014). Silicon multielectrode arrays have been used for several decades in animal experimentation (Kuperstein and Eichenbaum, 1985; Drake et al., 1988; Wise and Najafi, 1991; Csicsvari et al., 2003).

The pioneering study of Ward et al. (2009) compared several microelectrode arrays having fundamentally different configurations. The authors measured the electrical impedance, the charge capacity, the signal-to-noise ratio, the recording stability, and the elicited immune response in microelectrodes implanted in rats. They found a significant variability within and between the microelectrode types with no clear superior array.

Using commercially available intracortical electrodes, Karumbaiah et al. (2013) studied the effects of several two designs (cylindrical, planar), several sizes (15, 50, and 75 μ*m*) and tethering. Histological, transcriptomic, and electrophysiological analyses were performed at acute (3 day) and sub-chronic (12 week) time points. Quantitative analysis of histological sections indicated that Michigan 50 μ*m* and Michigan tethered electrodes induced significantly higher glial scarring, and lesser survival of neurons in regions of BBB breach when compared to microwire and Michigan 15 μ*m* electrodes. The findings were similar for the acute as well as the chronic time points. Over a period of 12 weeks, the electrophysiological assessment of electrode function yielded significantly better electrode performances for the microwire electrodes than for all the other designs. These results demonstrated that intracortical electrodes with smaller size, cylindrical shape, and without tethering cables produce significantly milder inflammatory responses when compared to large, planar and tethered electrodes.

These results contrast the ones obtained with Utah type of array. In our opinion, designs based on dense arrays without sufficient spacing between electrodes must be avoided.

## 6. Transient factors

### 6.1. Shaft buckling

Although matching the stiffness between the brain and an electrode shaft would have many advantages, it would result in a device that cannot be implanted unsupported because of shaft buckling. For reference, the critical buckling force depends also on the geometry (tensor of intertia, height) and the Young's modulus of the material. Therefore, the right choice of material, shape, and insertion speed are of paramount importance.

A probe shank made of polyimide rather than silicon can lead to more than one order of magnitude reduction of the forces necessary to bend the shank. Another promising approach is the use of composite materials integrating silicon with polyimide (Andrei et al., 2012a; Kim et al., 2014). Alternative methods are the incorpoartion in hard gelatin dissolving after implantation (Agorelius et al., 2015) or the backing of electrodes with biodegradable silk (Wu et al., 2015).

Mechanically-adaptive materials, rigid at the time of insertion but becoming more compliant after implantation under the influence of water and heat, might offer a solution to the conflicting requirements on stiffness to allow insertion and later mechanical tissue compatibility (Nguyen et al., 2014). Such implants have been evaluated chronically by Nguyen et al. (2014), who demonstrated that at 2, 8, and 16 weeks post-implantation, the compliant implants showed a significantly reduced neuroinflammatory response when compared to a stiff reference. The chronically implanted compliant electrode also yielded a more stable BBB. The data thus demonstrated that mechanically compliant intracortical implants can reduce the neuroinflammatory response in comparison to stiffer systems.

### 6.2. Acute vascular damage and hemorrhage

Insertion of the electrode leads to acute disruption of the BBB and hemorrhages from disrupted small brain blood vessels (Schmidt et al., 1993; Bjornsson et al., 2006). The disruption of BBB leads to the deposition of plasma proteins foreign to the CNS including albumin (40 mg/mL or 55%), globulins (10 mg/mL or 38%), fibrin/fibrinogen (3 mg/mL or 7%), thrombin, plasmin, complement, and red blood cells (hemosiderin) (recent review in Kozai et al., 2015).

The vascular damage is accompanied by fluid displacement, dragging of the blood vessels and eventual vessel severing. The most severe form of vascular damage is the vessel rupture, which is accompanied by hemorrhage (Bjornsson et al., 2006). For example, Ward et al. (2009) reports traumatic insertion of *Cyberkinetics* probes accompanied by hemorrhages. In their study the incidence of hemorrhages caused by insertion were for Cyberkinetis electrode—71%; for the Drexel University probes (Moxon et al., 2004)—60%; for the *NeuroNexus* probes—25%. The authors found also large variability of the histological responses, which they attributed to the occurrence of hemorrhages. Such damage causes BBB rupture, infiltration of leukocytes and platelets and extravasation of serum proteins, notably albumin, which can cause direct activation of astrocytes (Nadal et al., 1997) and microglia (Hooper et al., 2009). The variability in intracortical hemorrhaging resulting from microelectrode insertion was first demonstrated under two-photon imaging *in vivo* by Kozai et al. (2010). It was shown that penetrating a single large intracortical blood vessel resulted in significantly larger BBB bleeding areas compared with penetrating through many small capillaries. In *ex vivo* studies, compression and rupture of the transcranial BBB have been observed as far as 300 μ*m* from the probe during insertion (Bjornsson et al., 2006).

Hemorrhages have been shown to be particularly detrimental for long term recording (Stensaas and Stensaas, 1976; Turner et al., 1999; Grand et al., 2010). For example, Grand et al. (2010) report extensive neuronal loss around electrodes where bleeding did occur. Authors examined the short or long term effect of bleeding on neuronal and glial cell densities. Signs of serious bleeding were visible at both 1 and 12 weeks after surgery. Tissue around the electrode tracks was damaged and very few, if any, neurons or glial cells could be observed. In some cases, but not always, patches with severe neuron loss were detected near damaged tissue. Although neuronal cell bodies were observed in damaged tissue within 100μ*m* from the track, electron microscopy showed that their membranes were disrupted, and large cavities were present throughout damaged tissue. In contrast neuronal cell bodies and synapses could be found close (< 10 μ*m*) to the electrode track when bleeding could be avoided.

### 6.3. Current understanding of the immediate effects of implantation

The immediate effects are caused by the mechanical interaction of the device with the brain tissue. These are notably the vascular damage, the hemorrhage and the brain edema. Erythrocytic lytic products and notably hemoglobin are observed as early as one day after hemorrhage and can cause cellular injury through oxidative stress (Xi et al., 1998; Wu et al., 2002).

Within minutes of brain damage, microglial processes rapidly extend toward the injured site. The chemoattractive response is triggered by ATP released at the site of injury and the consequent activation of the purinergic receptors on microglia (Ohsawa and Kohsaka, 2011). In addition to these purinergic signals, other neuronal signaling molecules actively and negatively control microglial motility, which is important for regulating the functional activation of microglia in response to pathology. These still largely unknown parallel mechanisms can sometimes be destructive while at in other circumstances they may be constructive (Kigerl et al., 2009) acting on a different mechanisms.

Six hours after a probe insertion, 50% of the microglia at a distance of 130 μ*m* from the probe surface exhibit morphological characteristics of T-stage activation similar to that observed with laser-induced BBB damage (Kozai et al., 2012). At 6 h post-implantation, the transition band from inactive to active identification spanned between 70 and 210 μ*m* from the nearest probe surface. These results suggest a chemical gradient is immediately established following probe insertion.

Astrocytes, pericytes and the extracellular matrix (ECM) components provide both structural and functional support to the BBB. Astrocytes form borders (glia limitans) that separate neural from non-neural tissue along perivascular spaces, meninges and tissue lesions in the CNS. In healthy CNS tissue, these astrocyte borders form functional barriers that present molecular cues helping to restrict leukocyte access into brain parenchyma from adjacent non-neural tissues that exhibit high levels of leukocyte trafficking for immune surveillance (Owens et al., 2008; Sofroniew, 2015). Astrocites have been implied as both harmful and protective agents in traumatic conditions. For example, restoration of local ionic homeostasis, wound healing and limitation of inflammation have been proposed as beneficial functions of astrocytes (Silver and Miller, 2004; Sofroniew, 2009, 2015). On the other hand, astrocytes also have powerful pro-inflammatory potential (Sofroniew, 2015).

A live imaging study of astrocytic responses to acute injury revealed a selective juxtavascular proliferation and no major migration of astrocytes (Bardehle et al., 2013). This finding from two-photon imaging of stab brain injury is in sharp contrast with the initial *in vitro* studies which described opposing findings. Another surprise from this study is the suggestion that histologically observed increase in GFAP more likely results from overexpression rather than from a proliferation of astrocyites. This further confirms the role of GFAP as a marker of the underlying neuroinflammation process (and potentially the morphology of the astrocytic processes), and not for the number of astrocytes' bodies.

## 7. Persistent factors

Chronic interactions are important during the indwelling period. From these we will focus on the diffusion of substances and BBB damage. An important recent finding is the confirmation of the presence of bi-phasic nature of the reactive tissue response (Potter et al., 2012) with a highly variable acute stage that appears to respond well to various interventions (He et al., 2006; Purcell et al., 2009; Azemi et al., 2011). Acute factors can be addressed well *in vitro* by means of different types of surface functionalizations (discussion in Leach et al., 2010) however, animal studies have shown that engineered probes elicit similar host tissue response chronically, compared to their un-modified cohorts. Therefore, in our opinion, more focus is necessary on the persistent factors.

### 7.1. Micromotion

The magnitude of brain tissue micromotion relative to a brain implant and the impact of the mechanical stresses induced by such movements on the viability and function of the local brain tissue is frequently overlooked in the literature. Only relatively recently, Muthuswamy et al. (2003) and Gilletti and Muthuswamy (2006) have measured brain micromotion in animals. These authors have compared preparations with *dura mater* removed and with intact dura. In anesthetized rats, pulsatile surface micromotion due to respiratory pressure changes was in the order of 10–30 μ*m* while vascular pulsatility induced 2–4 μ*m* displacements. The presence of the dura significantly reduced the respiratory oscillations. Such microdisplacements of the brain against a relatively stationary electrode because of its tethering to the skull (Figure 1B) or simply because of its own mass inertia is bound to induce repeated stresses at the interface. In line with this theory, completely untethered implants have been shown to result in significantly smaller long-term scars than tethered ones (Kim et al., 2004; Biran et al., 2007; Thelin et al., 2011). In a model of chronic device implantation (Woolley et al., 2013), microglia counts changes and the presence of vimentin (demonstrating meningeal ingrowth and neovascularization in depth) suggest a mechanical force configuration similar to what is expected from Figure 1B. Similar results have been reported by Welkenhuysen et al. (2011) who demonstrated an increasing depth gradient of ED1 and GFAP immunoreactivity 6 weeks after implantation of a tethered device.

Motion of the brain in monkeys and humans is even more pronounced since beside the proportionally larger brain the subdural space is also much larger. For example, in monkeys the brain motion results in a periodic axial force of 5 mN with a frequency of 0.22 Hz (Hosseini et al., 2007).

Lee et al. (2005) simulated the micromotion around the implanted electrode using a Finite Elements Modeling (FEM) approach. The results indicate that the effects of micromotion and mechanical mismatch could broadly extend into the cortical tissue and that the shape of the electrodes and electrode arrays significantly impacts the strain produced. Mechanical forces in the NeuroNexus probes implantation have been simulated by Zhang et al. (2014). This study revealed that micromotion frequency has a great effect on the maximum von Mises stress, with higher frequencies being more harmful than lower frequencies in terms of the long-term stability of the electrode. When the frequency is 20 Hz, the stress almost reaches its maximum value which hardly increases at higher frequencies. Important mechanical interaction between an implant and brain tissue thus take place within the frequency range of vascular pulse and breathing movements. The results of the same study further indicated that enhancing the adhesion between the implant and the brain tissue can effectively decrease stress and strain.

Micromotions can result from physiologic movements including the important case when an implanted object having different density is present (Section 4.4). Physiologic movements have different components, notably body motion, pulse and respiration. In anesthetized rats the pulsatile surface micro-motion of the order of 10–30 μ*m* have been observed during respiration while components of 2–4 μ*m* are due to vascular pulsatility (Gilletti and Muthuswamy, 2006). The same authors observe that brain displacement values due to respiration are significantly lower in the presence of *dura mater* compared to those without dura. The impact of micro-displacement and hence of micro-forces is amplified if rigid electrodes are tethered to the skull because of the relative movements between the brain and the skull (Figure 1B). In line with this, completely untethered implants have been shown to result in significantly smaller long-term scars than tethered ones (Kim et al., 2004; Biran et al., 2007; Thelin et al., 2011). Tissue micro-motion induced stresses on the microelectrode constituted 12–55% of the steady-state stresses on the microelectrode on the day of implantation (*n* = 4), 2–21% of the steady-state stresses after 4 weeks of implantation (*n* = 4), and 4–10% of the steady-state stresses after 6–8 weeks of implantation (*n* = 7) (Sridharan et al., 2013).

### 7.2. Reactive oxygen species formation

Reactive oxygen species (ROS), such as peroxides superoxide, hydroxyl radical, and singlet oxygen, are involved in many physiological and pathological processes. ROS are present at moderate levels in every living cell where they fulfill vitally important functions under the conditions of redox homeostasis. Low amounts of ROS and reactive nitrogen species (RNS) are generated by different mechanisms in every cell and are important regulatory mediators in signaling processes (redox signaling) (reviews in Lehner et al., 2011; Hsieh and Yang, 2013). When the physiological balance between the generation and elimination of ROS is disrupted, oxidative/nitrosative stress with persistent oxidative damage of the organism occurs. There is a vast current literature on the oxidative stress and ROS effects, so we are not going to give additional details here. Interested readers could consult reviews in Abbott (2000); Réus et al. (2015); Hsieh and Yang (2013); Uttara et al. (2009).

The cerebral vasculature is particularly susceptible to the action of ROS (i.e., oxidative stress), which is of great importance since cerebral endothelial cells play a major role in the creation and maintenance of the BBB. ROS contribute to BBB disruption by several mechanisms: oxidative damage to cellular molecules (proteins, lipids and DNA), activation of matrix metalloproteinases (MMPs, review in Lehner et al., 2011), cytoskeletal reorganization, modulation of tight junction proteins and up-regulation of inflammatory mediators. ROS accumulation leads to oxidative stress on local cells.

BBB breakdown mediated by oxidative stress is a also common phenomenon in neurological diseases, including amyotrophic lateral sclerosis, multiple sclerosis and stroke (review in Obermeier et al., 2013). Such BBB dysfunction can result in an imbalance of ions, transmitters and metabolic products in the interstitial fluid, causing abnormal neuronal activity. This can explain elevation of neuronal firing observed in the first few weeks in the chronic studies. In the implantation setting ROS can be linked to the catalytic function of *Fe*^3+^ in the blood clot. As a model of such positive feedback loop can be regarded the cerebral ischemia-reperfusion injury, where ROS and RNS are also major players (Gu et al., 2011). Recent studies indicated that caveolin-1, a membrane integral protein located at caveolae, can prevent the degradation of tight junction proteins and protect the BBB integrity by inhibiting RNS production and MMPs activity. The interaction of caveolin-1 and RNS forms a positive feedback loop which provides amplified impacts on BBB dysfunction during cerebral ischemia-reperfusion injury. In a similar way in the chronic stage reactive oxygen species can self-perpetuate the chronic neuroinflammatory response by inducing secondary BBB breach and cellular damage. In support of this view we can highlight the study of Potter et al. (2013) who have shown some evidence that short-term attenuation of ROS accumulation and stabilization of BBB can result in improvements in neuronal viability around implanted intracortical microelectrodes up to 4 weeks, while also identifying potential therapeutic targets to reduce chronic intracortical microelectrode-mediated neurodegeneration. Therefore, it is likely that reduction of the accumulation of reactive oxygen species at the intracortical microelectrode-tissue interface could result in a direct improvement in BBB stability and neuronal health.

### 7.3. Persistent BBB leakage

Persistent BBB breach at the location of indwelling brain implants has been observed, and can have a negative effect on the function of chronic neural implants through recruitment of pro-inflammatory myeloid cells and increased presence of neurotoxic factors. Saxena et al. (2013) studied BBB function in chronic implantation conditions. Two widely used commercially available electrode arrays, the Neuronexus (planar) and microwire electrodes have been implanted in the cortex of adult rats. The electrode performances were correlated to a quantitative analysis of BBB breach and subsequent infiltration of myeloid cells and neurotoxic factors. The findings work demonstrated that functionally stable electrodes had less permeable BBB and lower transcript levels of those cytokines that exacerbate BBB permeability and neuroinflammation. Saxena et al. (2013) hypothesized that intracortical electrodes induced a chronic breach of the BBB which, in a positive feedback loop, leads to chronic inflammation, culminating in neurodegeneration and electrode failure. Potter et al. (2012) found two distinctly inverse multiphasic profiles for neuronal survival in device-implanted tissue compared to stab-injured animals. The marker for BBB leakage IgG was found around implanted devices for periods up to 16 weeks, while its amount around the stab injury was close to baseline. Karumbaiah et al. (2013) also observed that electrodes that fail chronically have a highly permeable BBB and therefore more active inflammatory cells and neurotoxic factors in their surroundings than electrodes that perform better.

### 7.4. Reaction-diffusion systems

**Figure 5:**
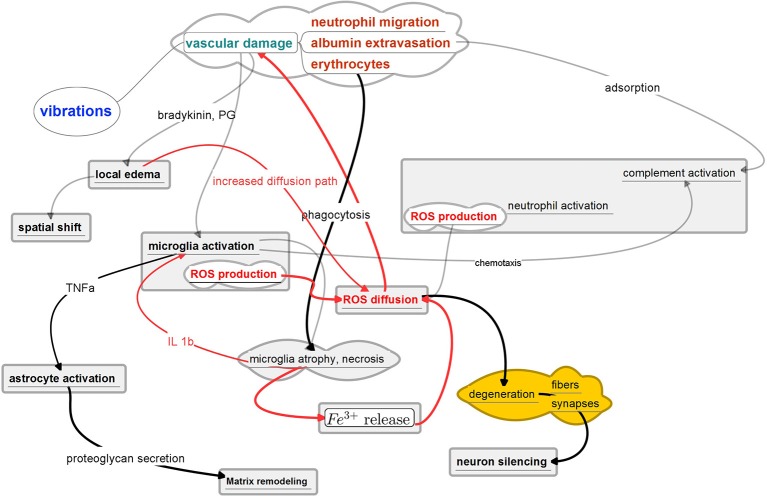
**Possible Motion - Reaction - Diffusion feedback loop in chronic implantation**. The positive feedback loops are indicated in red. Key element in the proposed mechanism is the sustained gradient of ROS. Depicted pathways are based on the recent findings of real-time immuno-chemical expression patterns (Karumbaiah et al., 2013; Saxena et al., 2013), two-photon imaging (Rosidi et al., 2011; Kozai et al., 2012) and detailed histological characterizations (Azemi et al., 2011; Potter et al., 2013; Woolley et al., 2013).

The interactions of persistent factors can be summarized in Figure 5. We hypothesize that micromotions caused by brain pulsations and inertial forces during movement lead to persistent disruption of BBB. The proposed mechanism starts by stretching of capillaries, which leads to albumin extravasation as well as macrophage, neutrophil and erythrocyte migration as first order events. Further, albumin adsorbs on the electrode shank which in turn activates the complement system (review in Gasque et al., 2000). Neutrophils, macrophages and microglia become activated. The adsorbed serum also releases chemo-attractants such as monocytes chemotactic protein-1 (MCP-1) and macrophage inflammatory protein (MIP-1) (Leach et al., 2010). Microglia and macrophages thus both migrate toward the shank of electrode and eventually adhere to the implant. These invasive macrophages are known to play an important role in stab wound reactions (Fujita et al., 1998). Activated neutrophils produce ROS which diffuses into the extracellular space. The macrophages and microglia phagocyte released erythrocytes which leads to even further ROS production and diffusion. If the amount of micro-hemorrhage (i.e., released *Fe*^3+^) in the tissue is small enough the process can be contained and eventually stopped as recently shown by Rosidi et al. (2011) who characterized the acute and chronic dynamics of cortical microhemorrhages by two-photon microscopy. These authors observed a rapid inflammatory response characterized by morphology changes in microglia/macrophages up to 200 μ*m* from the microhemorrhage as well as extension of cellular processes into the haemostatic clot. In localized microhemorrhages the extent of the inflammation did not trigger this neurotoxic response.

By contrast, in larger traumas, sustained production of ROS eventually leads to microglial atrophy and necrosis, explaining the dystrophic microglia found in the first days after implantation in failing tungsten wire microarrays (Prasad et al., 2012). These authors hypothesized that the break-up of microglial cytoplasm shortly after electrode implantation is the direct result of excessive oxidative stress that builds up at the implantation site due to an influx of various oxidative substances from the bloodstream, including free iron.

Macrophage/microglial necrosis in turn release debris containing *Fe*^3+^ in the extracellular space leading to further cycles of phagocytosis and necrosis, resulting in a self sustained process that generates a ROS gradient in the tissue. *O*_2_ species are light and volatile molecules that easily diffuse in the extracellular space. It is interesting to note that iron has been implied to affect neuronal activity by Codazzi et al. (2015). The iron-induced oxidative tone can, in physiological conditions, positively influence the calcium levels and thus the synaptic plasticity. On the other hand, an excess of iron, with the ensuing uncontrolled production of reactive oxygen species (ROS), is detrimental for neuronal survival. A protective mechanism can be played by astrocytes that, more resistant to oxidative stress, can uptake iron, thereby buffering its concentration in the synaptic environment. In Parkinson's disease, neuronal death and microglial ROS have been shown to subtend the chronic microglial neurotoxicity (Lull and Block, 2010). Diffusing ROS are toxic to the nerve fibers and synapses, leading to fiber atrophy and degeneration of synaptic bodies ending in the loss of neural activity.

The oxidative stress resulting from iron deposition in the extracellular matrix leads to the activation and upregulation of proinflammatory cytokines, such as interleukin (IL)-1β (overview in Kozai et al., 2015).

Through another branch of the local inflammatory pathways, microglia activation induces secretion of TNFα which in turn will lead to astrocyte activation as demonstrated by expression of GFAP. Activated astrocytes will secrete altered proteoglykans responsible for extracellular matrix remodeling. The cell adhesion molecules of the immunoglobulin superfamily (Ig-CAMs) have been shown to modulate long-term potentiation and long-term depression in the hippocampus (Dityatev et al., 2008).

Activated microglia can also induce dysfunction of the BBB by releasing IL-1β which upregulates MMP-9, a matrix metalloproteinase known to degrade the gap junction of BBB endothelial cells (Tian and Kyriakides, 2009). It has been demonstrated that continuous expression of IL-1β leads to continuous BBB leakage. On the other hand, continuous IL-1β expression, infiltration of leukocytes, and BBB leakage *in vivo* are not sufficient alone in causing neurotoxicity or neurodegeneration (review in Rothwell, 2003).

Moreover, prostaglandins and bradykinin released within the brain will induce local edema and increase of local blood flow resulting in a modifyed metabolic state of the local neurons (Wahl, 1985). Suggestively, the metalloproteinases have also been implicated in maintaining the BBB permeability in stroke (Rosenberg et al., 1998) by a mechanism where pericytes are implicated (Lai and Kuo, 2005; Zozulya et al., 2008). Primary CNS glia subjected to cyclic strain upregulate a host of proinflammatory cytokines and MMPs that could trigger tissue remodeling and interact with BBB (Karumbaiah et al., 2012).

Since all of the above factors are persistent then their action can lead to the appearance of steady state distributions of various diffusing substances surrounding the implanted electrodes.

Diffusion is a major transport mechanism in the brain. It is essential to the survival of neural tissue as seen with sub-retinal electrodes where the implant can clearly form a deleterious diffusion barrier (Peachey and Chow, 1999; Zrenner et al., 1999). Changes in the diffusion properties could, therefore, influence accumulation and removal of substances. The implant itself, scar tissue and the modified vascularization will profoundly modify the local chemical exchanges. Perforated implant structures can be a solution to alleviate this diffusion barrier effect.

**Figure 6:**
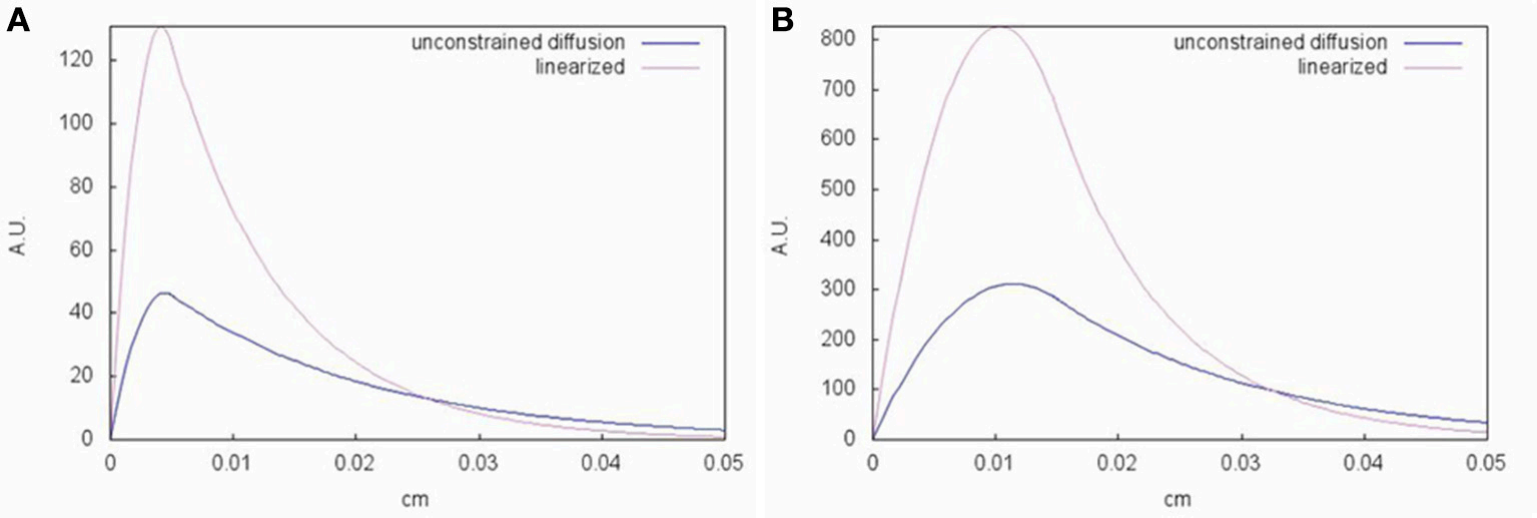
**Influence of source spatial extent on the steady-state distribution of diffusing species**. **(A)** Scar thickness is assumed to be 50μ*m*. **(B)** Scar thickness is assumed to be 150μ*m*. x-axis is shown in *cm*. y-axis shows normalized concentration of species with regards to source. The model assumes two compartments: scar with active diffusion source and tissue where the substance is also cleared by first order kinetics. *Linearized* refers to diffusion coefficient, which is linearly corrected for sub-diffusion according to Nicholson (2001); Syková and Nicholson (2008). A preprint version of the model is available in the ArXiv repository (Prodanov and Delbeke, 2015).

A diffusion model proposed by the present authors demonstrates that accumulation of species can occur at a distance from the electrode boundary and the implant geometry and scar tissue properties (i.e., tortuosity, fractional order, scar thickness) can influence such accumulation. A preprint version of the model is available in the Arxiv repository (Prodanov and Delbeke, 2015). Some simulation results are demonstrated in Figure 6.

Some authors speculated that the glial scar itself forms a diffusion barrier. This view is not supported well by observations. The scar exhibits thickness in the range of 50–300 μ*m*, which in normal circumstances appears to be insufficient to cause substantial *pO*_2_ drop, since this is maintained in the brain tissue by a homeostatic mechanism. Feedback mechanism exists, which senses the tissue *pO*_2_ and consequently transmits a signal to the vasculature such that cerebral blood flow is adjusted to maintain a constant *pO*_2_ (Leithner and Royl, 2014). On the other hand, if the blood oxygenation homeostasis is impaired such diffusion disturbances may gain impact.

Interestingly, it was shown that after 16 week implantation in the rat cortex thick permeable surface coatings, which served as diffusion sinks, significantly reduced the foreign body reaction compared to implants with no coating or with a thinner coating (Skousen et al., 2015). Animals with an increased level of chronic inflammation were associated with increased neuronal and dendritic, but not axonal loss. The neuronal and dendritic loss was more severe 16 weeks after implantation compared to 8 weeks after implantation, suggesting that the local neurodegenerative state is progressive. After 16 weeks, axonal pathology could be identified in the form of hyperphosphorylation of the protein tau in the immediate vicinity of the microelectrodes.

## 8. Conclusions

A chronically stable minimal distance from electrode contact to healthy neurones can be seen as the ultimate goal of the implantation of a neural interface device. Numerous aspects of living tissue reaction to the foreign body implantation put serious limitations on the achievable results. Many researchers, therefore, aim at investigating the mechanisms of that tissue reaction. The endeavor appears quite challenging because of the multiple timings of the events, the large number of cellular actors implicated, the complexities of the chemical cytokine pathways and the contribution of very different phenomena, such as sensitivity to strain/stress, neuroinflammation, the BBB concept, neuronal apoptosis, neuronal migration, diffusion barrier, fibrous deposition, etc…Various cytokines, neuropeptides and other chemicals have been identified as significant players. Their source and target of action in some cases have been identified. While some mechanisms are now clarified, we are still far from drawing the complete picture of the various pathways involved and their interactions most often remain obscure.

More and more publications suggest that chronic neuroinflammatory events and BBB breakdown are responsible for the mediocre performances of neural interfaces over long time periods. A self-sustained BBB breach plays a major role in this chronic state of affairs and is directly modulated by stress and strain. These mechanical factors are themselves continuously activated by micro-movements, inertial forces and gravity acting on the weight/density and stiffness of the implant with the contribution of device tethering and adhesion forces to the surrounding. Iso-dense electrodes with an overall stiffness similar to brain tissue would clearly represent a major step toward ideal neural implants. Combining such characteristics with realistic implantability remains a challenge.

Some very interesting experiments have been performed, limiting the tethering, using soft coatings and in particular variable stiffness polymers. The important question whether the chronic tissue reaction is self-sustained or it is only the result of the persistence of causal factors has not yet been resolved. In order to prove that the tissue reaction is not self sustained, we must eliminate completely each one of the numerous reaction triggering factors listed above. This is as yet an impossible task. The ideal implant made of material that is not detected as “foreign,” does not disturb diffusion, has the same stiffness and density as the brain, and can be implanted without tissue damage might never become feasible. On the other hand, tissue reaction results from nature's attempts to restore health after an aggression. The various mechanisms described in literature are thus not “destructive” by nature but can have very useful consequences. The glial scar, for example, can form a necessary mechanical interface between tissue and implant. Neovascularization can restore proper tissue maintenance. The fibrous encapsulation can stabilize the electrode-tissue interface. The main qualities of the implanted interface are thus better defined in terms of the target to electrode contact distance, a healthy target cells and long-term stability of the interface. These goals can often represent contradictory requirements and the ideal electrode interface should thus realize an optimal trade-offs for a given application.

Although much information has been gathered and is in process of accumulation we are still left with important gaps in our knowledge—to such a great degree that it is yet impossible to define parameters to monitor in the comparison of tissue reaction between studies. Also the understanding and the control of interactions between the numerous factors involved are by far not yet mastered. We still need substantial amount of basic research in simplified settings before integrated models can be elaborated to allow for a global design involving the tissue reaction as well as the implant in a single functional replacement or extension therapy. Clearly, the label “biocompatibility” can no longer be applied to such developments. We propose using the word *biocompatibility* exclusively in a regulatory context with direct reference to the absence of unacceptable biological damage.

Some research directions can be pointed out. These are, for example, measurements of mechanical interactions, transport and diffusion phenomena, exploration of the cellular interactions in different geometric and mechanical micro environments. As long as each of these aspects cannot be controlled, it seems impossible, to tease out the mechanisms of tissue healing and foreign body reaction *in vivo*.

## Author contributions

DP and JD participated in the conceptualizing and writing of the manuscript.

### Conflict of interest statement

The authors declare that the research was conducted in the absence of any commercial or financial relationships that could be construed as a potential conflict of interest.
